# A Case of Retrograde Treatment of a Ureteral Stone in a Retrocaval Ureter

**DOI:** 10.1089/cren.2018.0061

**Published:** 2018-12-17

**Authors:** Andrea Guttilla, Mario Fiorello, Vittorio Fulcoli, Alessandro Andrisano, Domenico Massari, Giuseppe Costa

**Affiliations:** Urology Department, Camposampiero Hospital, ULSS 6 “Euganea,” Camposampiero, Italy.

**Keywords:** retrocaval ureter, ureteroscopy, ureteral stone

## Abstract

***Background:*** Retrocaval ureter is a rare entity with a reported incidence of ∼1 in 1100 and a 2.8-fold male predominance. The course of the ureter could be classified, using an intravenous urography, as type 1 having S-shaped, fish-hook, or J-shaped retrocaval course or type 2 having sickle-shaped course. No case report describing retrograde endoscopic management of ureteral calculi in the presence of retrocaval ureter could be found in existing literature. We are presenting a case of type I retrocaval ureter with ureteral calculi and nonobstructive drainage, which was effectively managed by flexible ureteroscopy.

***Case Presentation:*** A 62-year-old Caucasian man presented with complaints of a renal colic. The patient was positive for a history of noninsulin-dependent diabetes and hypertension. A direct abdomen CT scan showed an 8 mm ureteral stone with suspected retrocaval course of right proximal ureter with no hydronephrosis. After informed consent, ureteroscopy was performed on the patient's right proximal ureter. No complications occurred intraoperatively and postoperatively. On follow-up of up to 3 months, patient was asymptomatic and direct abdomen CT scan showed normal kidney without hydronephrosis.

***Conclusion:*** In the presence of retrocaval ureter and associated ureteral calculi with a condition of nonobstructive drainage, retrograde ureteroscopy is a safe and optimal procedure.

## Introduction and Background

Retrocaval ureter is a rare entity with a reported incidence of ∼1 in 1100 and a 2.8-fold male predominance. The first observed case of retrocaval ureters was described by Hochstetter in 1893.^1^ Obstruction of the ureter is the predominant effect of an anatomical abnormality, since it is associated with an anomalous course of the ureter that could be posterior, medial, anterior, and finally lateral to the inferior vena cava (IVC). Using an intravenous urography (IVU), the course of the ureter could be classified as type 1 having an S-shaped, fish-hook, or J-shaped retrocaval course or type 2 having sickle-shaped course. Symptoms caused by the obstruction typically appear in the third or fourth decade of life. A surgical treatment is necessary in these cases and it consists of a mobilization of the ureter both above and below the retrocaval course and of an ureteroureterostomy. Sometimes, this vascular anomaly is not always associated with ureteral obstruction. The management of a patient with renal calculi along with a retrocaval ureter is hence laden with a dilemma: treat the renal calculus alone or also repair the ureteral anomaly. No cases report describing retrograde endoscopic management of ureteral calculi in the presence of retrocaval ureter could be found in existing literature. We are presenting a case of type I retrocaval ureter with ureteral calculi and nonobstructive drainage, which was effectively managed by flexible ureteroscopy.

## Case Presentation

A 62-year-old Caucasian man presented with complaints of a renal colic. The patient was positive for a history of noninsulin-dependent diabetes and hypertension. History of fever, hematuria and dysuria, and loss of weight were absent. Clinical examination of the abdomen was within normal limits. Complete laboratory evaluation, including urinalysis, complete blood picture, urea, creatinine, and electrolytes, showed a mild renal insufficiency (creatinine 2.1 mg/dL, glomerular filtration rate 72 mL/(min ·1.73 m^2^)).

**FIG. 1.**
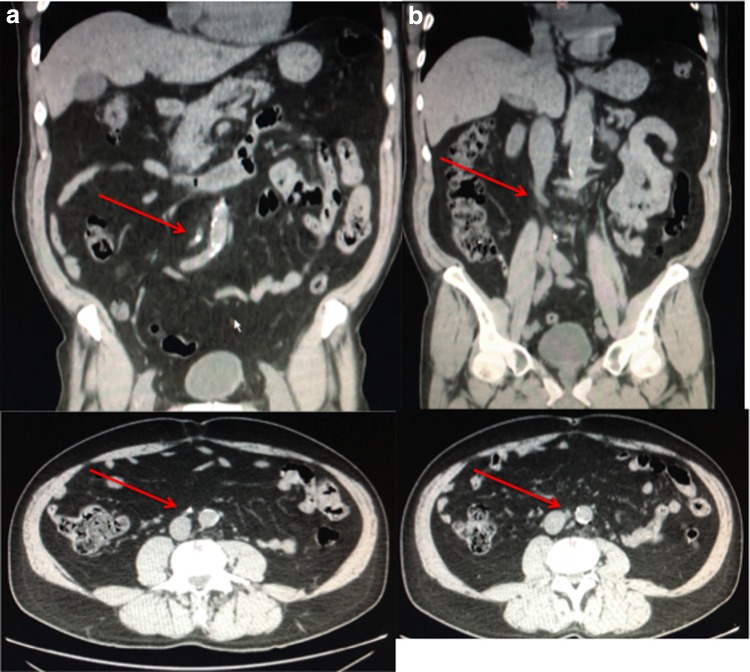
**(a)** Preoperative CT scan (*red arrow* shows the stone); **(b)** preoperative CT scan (*red arrow* shows the retrocaval ureter).

A direct abdomen CT scan (Fig. 1) showed an 8 mm ureteral stone with suspected retrocaval course of right proximal ureter with no hydronephrosis. After receiving the informed consent, ureteroscopy was performed on the patient's right proximal ureter.

**FIG. 2.**
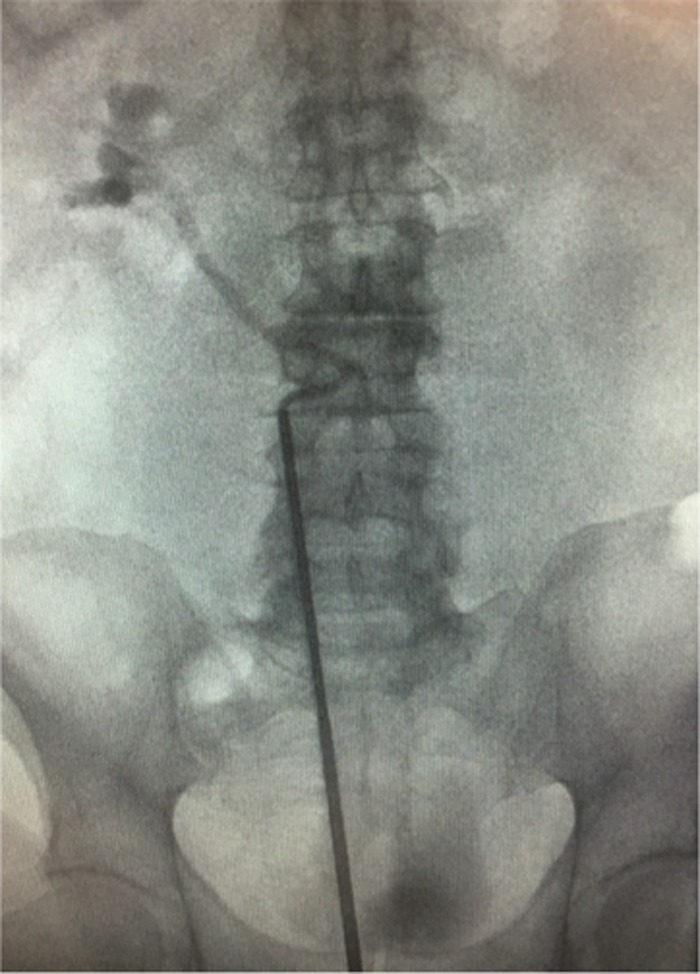
Right retrograde pyelography.

**FIG. 3.**
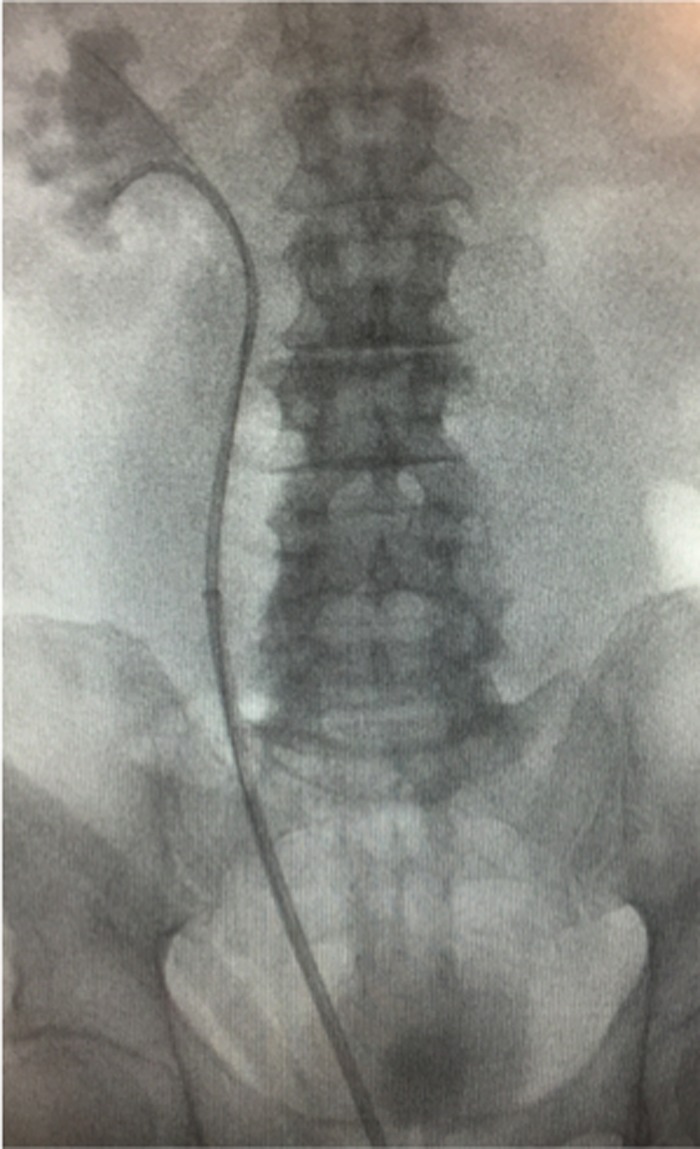
Retrograde flexible ureteroscopy.

After spinal anesthesia, a semirigid ureteroscopy after a right retrograde pyelography was performed (Fig. 2). The instrument was inserted just below the ureteral curve and a guidewire was placed (0.9 mm sensor guidewire) under fluoroscopic control in the right pelvis. Then the instrument was replaced with a flexible one (URF-P6; Olympus^®^) and without a ureteral sheath, to avoid an accidental perforation of the ureter at the retrocaval curve, a ureteroscopy was performed. The stone during the previous maneuvers was pushed up in the kidney. The stone was easily found in an inferior calix (Fig. 3) and taken off with a Zero Tip nitinol basket without intrarenal lithotripsy.

No complications occurred intraoperatively and postoperatively. After 3 months patient was asymptomatic and direct abdomen CT scan showed normal kidney without hydronephrosis.

## Discussion

Retrocaval ureter is a rare congenital anomaly 1 and it is caused by a congenital abnormality in the development of the vena cava. The most common theory of the development of such anomaly is that the subcardinal vein persists as the infrarenal IVC, thus crossing anterior to the midportion of the ureter and resulting in its circumcaval course.

Type I retrocaval ureter is more common and a marked hydronephrosis is seen in 50% of the patients.

In type II retrocaval ureter, the cross occurs at the level of the renal pelvis more frequently. There is lesser degree of hydronephrosis or none at all and the renal pelvis and upper ureter lie horizontally before encircling the vena cava in a smooth curve (sickle-shaped curve). The anomaly predominantly involves the right ureter.

Retrocaval ureter may be asymptomatic or discovered during radiological imaging for some other problems. However, some time during the excretory phase of CT scan entire ureter is not viewed because of pooling of contrast in the dilated renal pelvis and proximal ureter and this problem also occur in our case. Although not diagnostic, the appearance of retrocaval ureter on IVU is typical and is highly suggestive of the diagnosis. If the patient is symptomatic with documented subrenal functional obstruction, dismembered pyeloplasty is the gold standard treatment.

Surgical management is reserved for type 1 cases that are usually symptomatic. Patients with minimal caliceal dilation and without significant symptoms do not require surgery, but they need to be followed up.

Various reports are present in literature describing association of retrocaval ureter and renal calculi with their simultaneous surgical management. Simforoosh et al.^2^ have reported simultaneous treatment of renal stone and retrocaval ureter with laparoscopic technique.

No case reports describing management of renal calculi by ureteroscopy and its feasibility in the presence of retrocaval ureter could be found in existing literature. A case report described the feasibility of extracorporeal shock wave lithotripsy in the management of renal calculus in patients with retrocaval ureter^3^ The author was of the opinion that the placement of a Double-J stent probably altered the course of the ureter from long J shape to a less curved sickle shape, which offered little hindrance in clearance of stone fragments.

A case of an effective percutaneous nephrolithotomy treatment of a kidney stone in a type I retrocaval ureter was described by Prakash et al. in 2013 with effective results.^4^

Our patient had type I retrocaval ureter with concomitant ureteral calculi. Considering the nonobstructive drainage of the kidney, we treated only calculi.

## Conclusion

In the presence of retrocaval ureter and associated ureteral calculi with a condition of nonobstructive drainage, retrograde ureteroscopy is a safe and optimal procedure.

## Abbreviations Used

CT: computed tomography
IVC: inferior vena cava
IVU: intravenous urography
RGP: retrograde pyelography
